# Clinical Breast Cancer Registry of IR. Iran (CBCR-IR): Study Protocol and First Results

**DOI:** 10.34172/aim.2023.90

**Published:** 2023-11-01

**Authors:** Monireh Sadat Seyyedsalehi, Azin Nahvijou, Shaghayegh Haghjooy Javanmard, Mojtaba Vand Rajabpour, Amirreza Manteghinejad, Habibollah Pirnejad, Zahra Niazkhani, Arash Golpazir Sorkheh, Maryam Baniamer, Jamshid Anasari, Masoud Bahrami, Maryam Marzban, Atefeh Esfandiari, Seyedeh Masoumeh Ghoreishi, Novin Nikbakhsh, Yahya Baharvand Iran Nia, Shahram Ahmadi Somaghian, Mohammad Taghi Ashoobi, Fataneh Bakhshi, Alireza Ansari-Moghaddam, Mahdieh Bakhshi, Maryam Moradi Binabaj, Hassan Nourmohammadi, Ramesh Omranipour, Kazem Zendehdel

**Affiliations:** ^1^Department of Medical and Surgical Sciences, University of Bologna, Italy; ^2^Cancer Research Centre, Cancer Institute, Tehran University of Medical Sciences, Tehran, Iran; ^3^Applied Physiology Research Center, Isfahan Cardiovascular Research Institute, Isfahan University of Medical Science, Isfahan, Iran; ^4^Cancer Prevention Research Center, Omid Hospital, Isfahan University of Medical Sciences, Isfahan, Iran; ^5^Patient Safety Research Center, Clinical Research Institute, Urmia University of Medical Sciences, Urmia, Iran; ^6^Erasmus School of Health Policy & Management (ESHPM), Erasmus University Rotterdam, Rotterdam, The Netherlands; ^7^Nephrology and kidney Transplant Research Center, Clinical Research Institute, Urmia University of Medical Sciences, Urmia, Iran; ^8^Department of Surgery, School of Medicine, Kermanshah University of Medical Sciences, Kermanshah, Iran; ^9^Department of Chemical Engineering, Amirkabir University of Technology (Tehran Polytechnic), Tehran, Iran; ^10^Depertment of Radiation Oncology, Arak University of Medical Sciences, Arak, Iran; ^11^Arak University of Medical Sciences, Arak, Iran; ^12^Clinical Research Development Center, The Persian Gulf Martyrs, Bushehr University of Medical Science, Bushehr, Iran; ^13^Statistical Genetics Lab, QIMR Berghofer Medical Research Institute, Brisbane, QLD, Australia; ^14^Department of Health Policy and Management, School of Medicine, Bushehr University of Medical Sciences, Bushehr, Iran; ^15^Cellular and Molecular Research Center, Health Research Institute, Babol University of Medical Sciences, Babol, Iran; ^16^Cancer Research Center, Health Research Institute, Babol University of Medical Sciences, Babol, Iran; ^17^Department of Internal Medicine, Lorestan University of Medical Sciences, Khorramabad, Iran; ^18^Shahid Rahimi Hospital, Lorestan University of Medical Sciences, Khorramabad, Iran; ^19^Razi Clinical Research Development Unit, Guilan University of Medical Sciences, Rasht, Iran; ^20^Guilan Road Trauma Research Center, Guilan University of Medical Sciences, Rasht, Iran; ^21^Social Determinants of Health Research Center, School of Health, Guilan University of Medical Sciences, Rasht, Iran; ^22^Health Promotion Research Center, Zahedan University of Medical sciences, Zahedan, Iran; ^23^Non-Communicable Diseases Research Center, Sabzevar University of Medical Sciences, Sabzevar, Iran; ^24^Department of Internal Medicine Shahid Mostafa Khomeini Hospital, Ilam, Iran; ^25^Department of Surgical Oncology, Cancer Institute, Tehran University of Medical Sciences, Tehran, Iran; ^26^Cancer Biology Research Center, Cancer Institute, Tehran University of Medical Sciences, Tehran, Iran

**Keywords:** Breast cancer, Health policy, Hospital, Quality indicator, Registry

## Abstract

**Background:**

Breast cancer (BC), as a significant global health problem, is the most common cancer in women. Despite the importance of clinical cancer registries in improving the quality of cancer care and cancer research, there are few reports on them from low- and middle-income countries. We established a multicenter clinical breast cancer registry in Iran (CBCR-IR) to collect data on BC cases, the pattern of care, and the quality-of-care indicators in different hospitals across the country.

**Methods:**

We established a clinical cancer registry in 12 provinces of Iran. We defined the organizational structure, developed minimal data sets and data dictionaries, verified data sources and registration processes, and developed the necessary registry software. During this registry, we studied the clinical characteristics and outcomes of patients with cancer who were admitted from 2014 onwards.

**Results:**

We registered 13086 BC cases (7874 eligible cases) between 1.1.2014 and 1.1.2022. Core needle biopsy from the tumor (61.25%) and diagnostic mammography (68.78%) were the two most commonly used diagnostic methods. Stage distribution was 2.03% carcinoma in situ, 12% stage I, 44.65% stage II, 21.32% stage III, and 4.61% stage IV; stage information was missing in 1532 patients (19.46%). Surgery (95.01%) and chemotherapy (79.65%) were the most common treatments for all patients.

**Conclusion:**

The information provided by this registry can be used to evaluate and improve the quality of care for BC patients. It will be scaled up to the national level as an important resource for measuring quality of care and conducting clinical cancer research in Iran.

## Introduction

 Breast cancer (BC), as a significant global health issue, is the most diagnosed cancer in women with an estimated 2.3 million new cases annually, representing 25% of all cancer diagnoses among women.^1^ BC is one of the most diagnosed cancers in the low-, and middle-income countries, including Iran.^2-5^ Since the incidence and mortality rates of BC are increasing, almost all countries are facing an economic burden due to BC.^2,6,7^ However, the lifetime risk of developing BC differs by country and ethnicity due to exposure to different risk factors. Various factors determine the incidence and mortality rates of BC in different countries, including economic development, environmental factors, and ethnicity.^6^ When comparing data from developed and developing countries, differences are observed in BC incidence and mortality rates. Comparatively, developed countries display high incidence and low mortality rates of BC, whereas developing countries display low incidence and high mortality rates. The disease is most reported in Western Europe and the United States, while it is least reported in Africa and Asia, which may reflect a false prevalence.

 BC prevention, lack of awareness and screening protocols, lack of or limited access to diagnostic centers in rural areas for early detection, lower standards of healthcare facilities, and improper management of diagnosed cases could have significant impact on treating this disease.^7-10^ Hence, the efforts of the World Health Organization (WHO) are focused on three main pillars within the Global Breast Cancer Initiative (GBCI), namely: 1) health promotion and early detection, 2) reduced delay to health system access, and 3) comprehensive BC management, particularly where cancer programs are often inaccessible and under resourced.^11^ For the sake of the three main GBCI programs, registries can serve as an integral part for monitoring the programs and providing evidence for informed decision makings.^12,13^ Furthermore, registries can affect other approaches, such as convening stakeholders and developing a platform for action and operational guidance.^12^ In this regard, hospital-based cancer registries have collected different data elements about BC patient management, such as diagnosis, treatment, follow-up, and quality indicators.^14,15^

 Similar to most other countries, BC is the most common cancer in Iran and annually more than 17 000 new BC cases are diagnosed.^1^ It is expected that the incidence rate of BC will be doubled in a decade.^9^ Therefore, early detection and proper management of BC is a growing public health issue. We established a multicenter clinical breast cancer registry in Iran (CBCR-IR) to collect data about BC cases, the pattern of care, and the quality-of-care indicators in different hospitals across the country. The results of this registry provide evidence for health policy making and for interventions to improve diagnostic and therapeutic procedures in hospitals, at local and national levels. In this paper, we aimed to introduce the CBCR-IR, its study protocol, and its early descriptive results during the first five years of the registry implementation.

## Objectives

 The main objective of this registry was to collect the hospital-based data and to evaluate the quality of BC care in Iran and compare the quality-of-care indicators in different settings in Iran. The specific objectives of the registry are:

- To evaluate the patterns of care and benchmark the patient outcomes in different regions,
- To evaluate the effectiveness of diagnosis and treatment rates over time,
- To improve the quality of BC care,
- To develop a database for clinical and epidemiological research,
- To develop a basis for professional education.

## Materials/Patients and Methods

###  History of the CBCR-IR Registry Design

**Figure 1:**
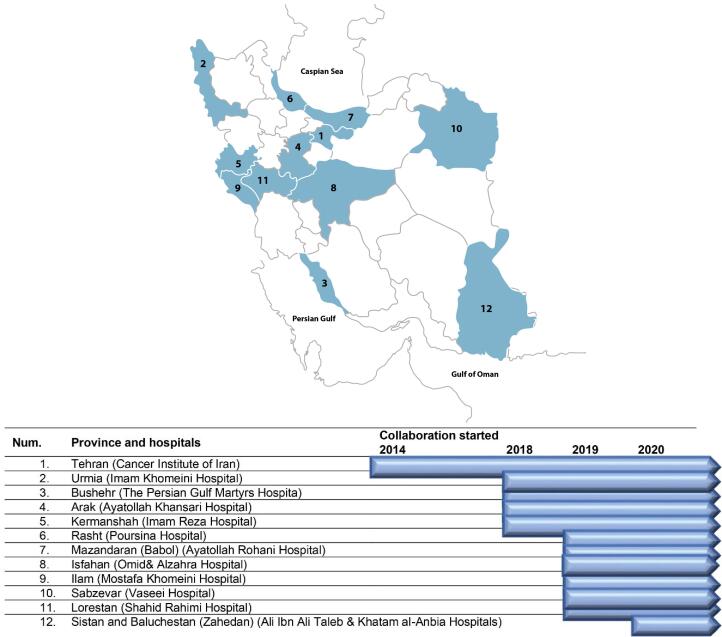
Centers Involved and Structure of Clinical Breast Cancer Registry of IR. Iran (CBCR-IR)

 For the first time in 2012, international experts recommended establishing clinical/hospital-based cancer registries in Iran.^16^ The clinical cancer registry was established in 2014 in the Cancer Institute of Iran in the form of a hospital cancer registry that prioritized data on four common cancer sites in Iran, including breast, esophagus, stomach, and colorectal cancers. The details about the implementation steps and the results of the pilot phase have been published elsewhere.^17^ In 2018, following the successful experience of hospital cancer registry in the Cancer Institute of Iran, we designed and established a national collaboration network of oncology centers and hospitals across the country to join the registry (a multicenter CBCR-IR). Currently, we are collaborating with 10 provinces (including 16 hospitals) (Figure 1).

###  Establishment of the Minimum Dataset and the Software

 During the pilot phase, a primary minimum dataset was developed using different international sources such as the International Classification of Diseases, 3rd edition (ICD-O-3),^18^ the 2016 Revision of Facility Oncology Registry Data Standards (FORDS),^19^ and the review of the international literature. The minimum dataset was further adjusted by a panel of experts including oncologists, surgeons, and epidemiologists. After the pilot phase of using the minimum dataset, it was updated and included some risk factors related to patient outcomes, comorbidities, and biopsy information. We also included both clinical and pathological staging of BC patients. The registry currently collects about 200 variables in different domains (Box 1). We adapted a web-based and open-source software called the District Health Information Software (DHIS2) for online data collection.^20^

**Box 1. The minimum dataset of the Clinical Breast Cancer Registry of IR. Iran (CBCR-IR)**

** Identifying information**

Hospital name

Demographics (gender, nationality, etc.)

Contact details

** Risk factors**

Smoking, alcohol, opium, and hookah use

Menopause status and pregnancy at the time of diagnosis

History of breast cancer or other cancers

History of radiation on chest or neck

History of breast or ovarian cancer among the first- and second-degree relatives

** Comorbidity**

Cardiovascular diseases

Kidney and liver diseases

Chronic lung diseases

Cancers

Diabetes and overweigh

Brain and neurologic diseases

** Biopsy and imaging information**

Fine-needle aspiration (FNA), Core needle biopsy (CNB)

Mammography, sonography (breast, abdominal and pelvic), MRI (breast, brain), CT scan (chest wall, abdominal, and pelvic)

** Staging (clinical - pathologic) and prognostic factors**

Cancer identification

Tumor information

Lymph node information ‎

** Immunohistochemical factors**

Estrogen Receptor (ER), Progesterone Receptor (PR), Human epidermal growth factor receptor 2 (HER2), Ki67, P53

** Treatment**

Surgery

Chemotherapy (neoadjuvant and adjuvant)

Radiotherapy (neoadjuvant and adjuvant)

Hormonotherapy

Targeted therapy

** Follow-up**

Vital status

Cause of death

Presence of local recurrence or distant metastasis

** Type of record dates**

Date of birth

Date of diagnosis

Date of biopsy sampling and imaging processes

Date of treatments

Date of last contact

Date of death

Date of local recurrence or distant metastasis

###  Structure and Organization

**Figure 2:**
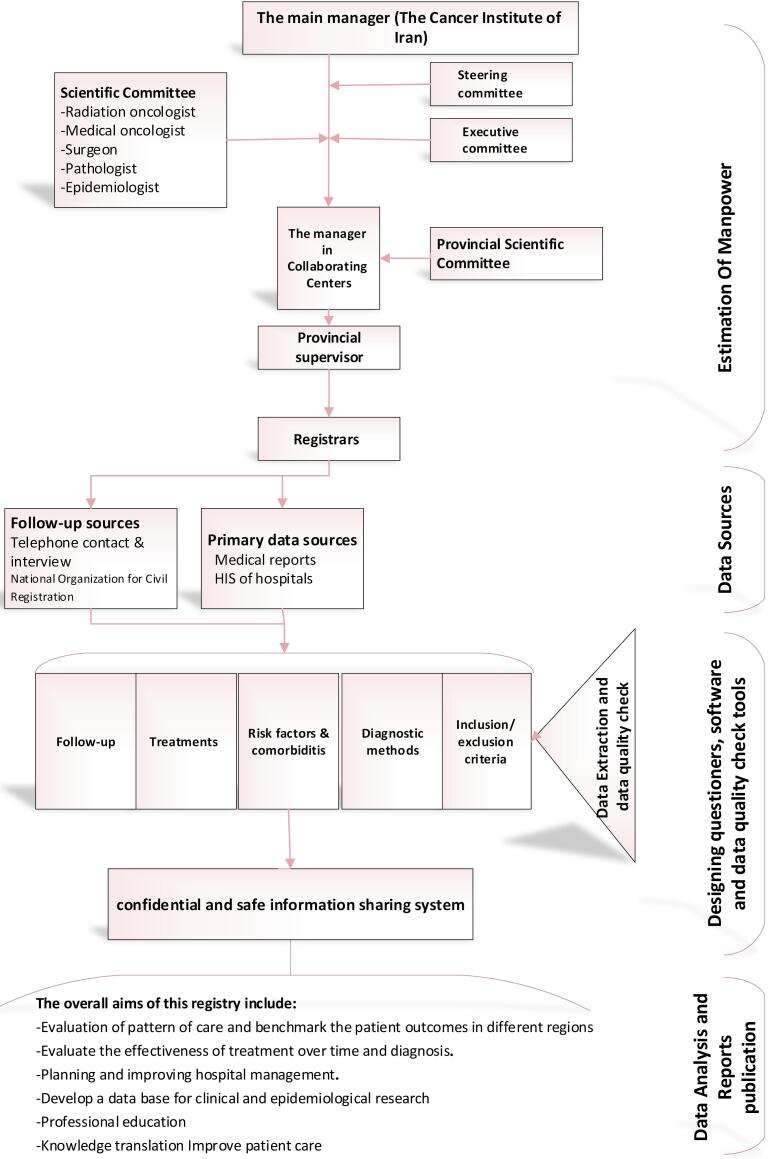
Flowchart of Patient Identification and Data Collection for the Clinical Breast Cancer Registry of IR. Iran (CBCR-IR)

 So far, 16 hospitals from 12 provinces across the country have been collaborating in the CBCR-IR network (Figure 1). The Cancer Institute of Iran is hosting and coordinating the registry network. In each collaborating center, a co-principal investigator (Co-PI) supervises a registry team to collect and monitor the quality of data of the registry in collaboration with the Cancer Institute (Figure 2). The recruitment of new centers is an ongoing process, and the number of centers is increasing. The processes for recruitment include evaluation of the readiness of a center, performing a pilot study and signing a collaboration agreement with the registry, and participation in a training session. Representatives from the participating centers are the members of the steering committee of CBCR-IR which act as the governing body for the registry.

###  Selection of Hospitals or Centers/Provinces

 The process of province selection includes five steps, namely: 1) sending a call for collaborating centers; 2) completing application and feasibility forms by the volunteering centers; 3) implementation of pilot phases in volunteer centers; 4) reviewing the results obtained by the Cancer Institute of Iran, selecting eligible centers; 5) signing an agreement between the main center and collaborating centers.

 Each collaborating center establishes its organizational structure similar to the structure of the main center except on a smaller scale.

###  Education and Training

 Two types of training courses were held, including in-person workshops at the beginning of activities in each center and periodic online workshop. In addition, a forum supports the registrars across the country and provides answers and explanations for the problems faced during the routine work.

###  Data Collection Process

 The primary product of the cancer registry is reliable data on cancer cases. Various programs and major decisions are based on these data. Therefore, selecting the most appropriate method for data collection and determining the structure for quality control of data is critical for a cancer registry. It is imperative to note that a clinical cancer registry is structured by collecting data from different hospitals and health centers with an expansive range of differences in geographical location, access to diagnostic equipment, specialist resources, and financial constraints. As part of our clinical cancer registry, 12 provinces and 16 hospitals and health centers were included. These centers varied in terms of the number of patients treated as well as other parameters. Thus, the design and implementation of the project required a variety of management programs and flexibility to coordinate different departments.

 The essential source of BC registry data items is medical reports (both inpatient and outpatient). Other sources for data collection were also used, including hospital information systems, and pathology and imaging reports. The centers contact patients and collect materials and information from patients or their next of kin. We also collect documents and follow-up information from the National Organization for Civil Registration, and actively through telephone interviews with patients or their caregivers.

###  Inclusion and Exclusion Criteria

 We set the commencing date of the registry on January 1, 2014, and BC patients’ data was registered if their diagnosis was made after this date. The starting date of the registry for collaborating centers varied across different centers based on availability of the data. Each center recruited its patients if they had a diagnosis of BC and received one of their initial treatment procedures in that specific hospital. BC morphologies other than adenocarcinoma such as melanomas, sarcomas, and lymphomas were excluded from the CBCR-IR.

###  Limitation and Implementation Challenges

 Our cancer registries had several limitations in terms of data collection and accuracy that needed to be addressed. These included 1) lack of access to old patient records, particularly those from the past few years, 2) lack of a specific and regulated structure for collecting diagnostic and therapeutic data on patients referred to medical centers by medical staff, 3) absence of electronic record systems, 4) illegibility of patients’ paper-based history records, 5) lack of access to patients for follow-up due to incorrect contact information, death or change in residence or unwillingness to cooperate because of dissatisfaction with the medical services provided, 6) lack of sufficient registrar training, 7) the need to update the registration guide and to hold continuous training sessions on how to access the right resources and gather quality information (Table 1).

**Table 1:** Data Source Used by Different Centers for Collection of Patient Data in the Clinical Breast Cancer Registry of IR. Iran (CBCR-‎IR)

**Center Name**	**Medical Report**	**Telephone Contact**	**In-person Interview**	**Link with Other Centers or Provinces for Data Collection**
Tehran (Cancer Institute of Iran)	Yes	Yes		
Urmia (Imam Khomeini Hospital)	Yes			
Bushehr (The Persian Gulf Martyrs Hospital)	Yes	Yes		Yes
Arak (Ayatollah Khansari Hospital)	Yes			
Kermanshah (Imam Reza Hospital)	Yes			
Rasht (Poursina Hospital)	Yes	Yes		
Mazandaran (Babol) (Ayatollah Rohani Hospital)	Yes			
Isfahan (Omid& Alzahra Hospital)	Yes	Yes		
Ilam (Mostafa Khomeini Hospital)	Yes			
Sabzevar (Vaseei Hospital)	Yes			
Lorestan (Shahid Rahimi Hospital)	Yes	Yes	Yes	
Sistan and Baluchestan (Zahedan) (Ali Ibn Ali Taleb & Khatam al-Anbia Hospitals)	Yes	Yes		

###  Quality Control Process

 In terms of completeness and validity, our quality control team (including an oncologist expert and epidemiologist at the cancer institute) uses several methods to validate our registry data through: 1) reviewing 10 percent of randomly selected data from each year’s registered cases by trained registrars and providing feedback to the principal investigators (PIs) and registrars in each study site and 2) cross-validating with other sources of data, e.g. by contacting patients or their caregivers and/or using data from other databases (e.g. causes of death registry, operation lists, etc) in order to complete the missing information and continuously monitor data validity. Centers are allowed, if necessary, to interview patients face-to-face and collect missing data items.

###  Ethical Considerations and Privacy Issues with Secondary use of Data

 We obtained ethics approval from Tehran University of Medical Sciences (TUMS) (Ethical Code: IR.TUMS.VCR.REC.1398.1015). Access to the data processing software is role-based and limited to people who are introduced by the manager of each center. Registrars and managers access the registry system using safe passwords.

 The CBCR-IR’s online web-tool has various role-based access levels managed by the director at the Cancer Institute of Iran; for example, registrars can only import and edit data but cannot delete or export data. Moreover, each supervisor in different centers can export their own data and cannot access other centers’ data.

 Furthermore, registrars and all team members have been trained to preserve the confidentiality of patient data and consider the ethical principles of this registry. In addition, we have developed a data sharing guideline and defined the roles and responsibilities for the registry teams and investigators requesting the data. Data are anonymized before sharing with researchers. All patient information is periodically backed up by the main center and the software server is protected by the Tehran University of Medical Science.

## Results

 From a total of 13 086 cases who were admitted to our network’s hospitals between January 1, 2014 to January 1, 2022, 7874 eligible BC patients were included in our analysis. The patients’ mean age at the time of diagnosis was 49 ± 13.60 years. Only 271 (3.44%) cases had a screening mammography (Table 2). The diagnostic methods were core needle biopsy from the tumor (61.25%) and diagnostic mammography (68.78%).

**Table 2:** Diagnostic Methods of Breast Cancer Currently Included in the Clinical Breast Cancer Registry of IR. Iran (CBCR-IR)

		**Province, No. (%)**												
		**Total**	**Center** 1: Tehran*	**Center** 2: Urmia***	**Center** 3:Bushehr*	**Center** 4: Arak***	**Center** 5: Kermanshah***	**Center** 6: Rasht**	**Center 7: Babol**	**Center 8:** Isfahan*	**Center** 9: Ilam***	**Center** 10:Sabzevar***	**Center 11:** Lorestan*	**Center** 12: Zahedan*
Overall		7874	3244 41.20%	396 5.03%	225 2.86%	266 3.38%	309 3.92%	411 5.22%	198 2.51%	2390 30.35%	48 0.61%	101 1.28%	159 2.02%	127 1.61%
Diagnostic methods	Mammography (screening)	271 3.44%	99 3.05%	10 2.53%	8 3.56%	15 5.64%	0	3 0.73%	2 1.01%	121 5.06%	2 4.17%	2 1.98%	9 5.66%	0
	Mammography (diagnostic)	5416 68.78%	2444 75.34%	109 27.53%	189 84%	119 44.74%	85 27.51%	280 68.13%	31 15.66%	1947 81.46%	5 10.42%	17 16.83%	127 79.87%	63 49.61%
	Breast MRI	1297 16.47%	870 26.82%	5 1.26%	6 2.67%	3 1.13%	7 2.27%	177 43.07%	0	180 7.53%	1 2.08%	2 1.98%	8 5.03%	38 29.92%
	Core needle biopsy (tumor)	4823 61.25%	2249 69.33%	101 25.51%	95 42.22%	73 27.44%	48 15.53%	9 2.19%	84 42.42%	1952 81.67%	5 10.42%	24 23.76%	129 81.13%	54 42.52%
	FNA (lymph node)	1224 15.54%	801 24.69%	6 1.52%	11 4.89%	6 2.26%	55 17.80%	228 55.47%	7 3.54%	72 3.01%	5 10.42%	1 0.99%	10 6.29%	22 17.32%
	Open biopsy	1188 15.09%	501 15.44%	174 43.94%	54 24%	1 0.38%	121 39.16%	104 25.30%	139 70.20%	21 0.88%	0	7 6.93%	16 10.06%	50 39.37%

Numbering centres according to the place in the map (Figure 1). * Information was collected using medical reports and telephone or in-person interview with patients ** Information was collected using telephone interviews with patients without access to medical reports *** Information was collected using medical reports

 Stage distribution was 2.03% carcinoma in situ, 12% stage I, 44.65% stage II, 21.32% stage III, and 4.61% stage IV; stage information was missing in 1532 patients (19.46%) (Table 3). On the basis of immunohistochemical information, 4409 (55.99%) of patients were estrogen receptors/ progesterone receptor (ER/PR) positive and 1725 (21.91%) were human epidermal growth factor receptor (HER2) positive. However, the ER/PR and HER2 status were missing in 24.55% and 25.22%, respectively (Table 3).

**Table 3:** Basic Characteristics of Breast Cancer Patients Currently Included in the Clinical Breast Cancer Registry of IR. Iran (CBCR-IR)

		**Province, N (%)**												
		**Total**	**Center** 1: Tehran*	**Center** 2: Urmia***	**Center** **3:** Bushehr*	**Center** 4: Arak***	**Center** 5: Kermanshah***	**Center** 6: Rasht**	**Center 7: Babol**	**Center 8:** Isfahan*	**Center** 9: Ilam***	**Center** **10:** Sabzevar***	**Center 11:** Lorestan*	**Center** 12: Zahedan*
Overall		7874	3244 41.20%	396 5.03%	225 2.86%	266 3.38%	309 3.92%	411 5.22%	198 2.51%	2390 30.35%	48 0.61%	101 1.28%	159 2.02%	127 1.61%
Staging at diagnosis (clinical /pathologic)	0	160 2.03%	124 3.82%	7 1.77%	0	1 0.38%	0	1 0.24%	0	15 0.63%	0	0	0	0
	I	939 11.93%	395 12.18%	33 8.33%	42 18.67%	26 9.77%	24 7.77%	0	23 11.62%	358 14.98%	4 8.33%	10 9.90%	35 22.02%	1 0.79%
	II	3201 40.65%	1386 42.73%	120 30.30%	86 38.22%	144 54.14%	75 24.27%	6 1.46%	81 40.91%	1,160 48.54%	11 22.92%	49 48.51%	75 47.17%	8 6.30%
	III	1,679 2132%	650 20.04%	152 38.38%	49 21.78%	59 22.18%	56 18.12%	1 0.24%	49 24.75%	569 23.81%	9 18.75%	29 28.71%	39 24.53%	17 13.39%
	IV	363 4.61%	154 4.75%	16 4.04%	10 4.44%	8 3.01%	42 13.59%	0	9 4.55%	89 3.72%	1 2.08%	8 7.92%	10 6.29%	16 12.60%
	Unknown	1532 19.46%	535 16.49%	68 17.17%	38 16.89%	28 10.53%	112 36.25%	403 98.05%	36 18.18%	199 8.33%	23 47.92%	5 4.95%	0	85 66.93%
ER /PR receptor	Positive	4409 5599%	1866 57.52%	130 32.83%	150 66.67%	162 60.90%	143 46.28%	8 1.95%	98 49.49%	1,641 68.66%	10 20.83%	64 63.37%	110 69.18%	27 21.26%
	Negative	1532 19.46%	601 18.53%	53 13.38%	53 23.56%	56 21.05%	48 15.53%	1 0.24%	21 10.61%	612 25.61%	7 14.58%	27 26.73%	37 23.27%	16 12.60%
	Unknown	1933 24.55%	777 23.95%	213 53.79%	22 9.78%	48 18.05%	118 38.19%	402 97.81%	79 39.90%	137 5.73%	31 64.58%	10 9.90%	12 7.55%	84 66.14%
HER2 receptor	Positive	1725 21.91%	687 21.18%	59 14.90%	70 31.11%	44 16.54%	124 40.13%	2 0.49%	31 15.66%	597 24.98%	12 25%	21 20.79%	51 32.08%	27 21.26%
	Negative	3661 46.49%	1490 45.93%	109 27.53%	112 49.78%	146 54.89%	65 21.04%	7 1.70%	83 41.92%	1,492 62.43%	7 14.58%	61 60.40%	70 44.03%	19 14.96%
	borderline	502 6.38%	261 8.05%	10 2.53%	23 10.22%	28 10.53%	1 0.32%	0	6 3.03%	146 6.11%	0	6 5.94%	21 30.21%	0
	Unknown	1986 25.22%	806 24.85%	218 55.05%	20 8.89%	48 18.05%	119 38.51%	402 97.81%	78 39.39%	155 6.49%	29 60.42%	13 12.87%	17 10.69%	81 63.78%

Centre numbering according to the location in the map (Figure 1). * Information was collected using medical reports and telephone or in-person interviews with patients. ** Information was collected using telephone interviews with patients without access to medical reports. *** Information was collected using medical reports.

**Figure 3:**
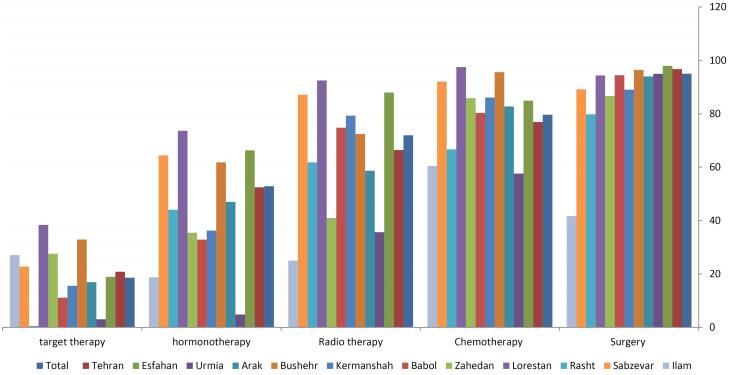
Treatment Methods of Breast Cancer Patients Currently Included in the Clinical Breast Cancer Registry of IR.Iran (CBCR-IR)

 Surgery (95.01%) and chemotherapy (79.65%) were the most common treatments for all patients (Figure 3). Only about 17% of patients underwent neoadjuvant chemotherapy. We also found that 66.43% of patients received adjuvant radiotherapy. The proportion of hormone therapy and targeted therapy in our study population was 52.88% and 20.81%, respectively.

## Discussion

 This program provides a valuable database about BC patient care from different geographical areas in Iran. It includes more than 200 variables and collects high quality data about demographics, risk factors, diagnosis, treatments, and follow-ups. This registry has registered more than 8000 BC patients across the country and provides an opportunity to conduct several studies about BC care and to determine the status of the care indicators of BC care in Iran overall and by hospitals located in different parts of the country. The registry makes it possible to compare hospitals and monitor the improvement after interventions in the patient care in each participating center and the country overall. As a result, policy makers will receive ongoing feedback that will help reduce inequalities across hospitals, improve tailored treatments, and increase compliance with national guidelines.

 Clinical performance and outcomes can be measured and tracked using quality indicators (QIs), which are standardized, evidence-based measures of quality of care. A list of 33 benchmark QIs was proposed by the European Society of Breast Cancer Specialists (EUSOMA) in 2010 and updated in 2017 to allow standardized auditing and quality assurance of care.^21^ Clinical cancer registries as a source of information about steps of diagnosis and treatment can help to determine and publish QIs that are being used in a variety of medical studies, and they provide an ideal infrastructure for estimating QIs regularly and comparing IQs across hospitals that may facilitate changes in guidelines.^22^ For example, studies published recently have shown that women undergoing breast conserving therapy have a better survival rate than those undergoing mastectomy.^21-27^ Quality care assessment is common in developed nations but is very rare in developing countries. Recently, a study in Morocco reported the result of breast QIs in that country.^28^ Our registry provides access to large datasets from several hospitals, which can be used to evaluate the quality-of-care levels across Iran.

 In addition to evaluating standards and guideline adherence, it is crucial to determine limitations and correct weaknesses in the diagnosis and treatment processes. One of the most critical sections relates to improving the infrastructure and access to essential cancer care services, which is expected to improve patient outcomes and reduce cancer incidence. A study showed that Eastern Mediterranean Regional (EMRO) countries, such as Iran, encounter varying conditions in terms of access to diagnostic equipment, such as computerized tomography (CT) scanners, magnetic resonance imagines (MRIs), and positron emission tomography (PET) scanners.^29^ In the EMRO region, our CBCR is the largest database, which can answer a wide range of questions regarding quality of care and access to health services. Also, governments with a focus on CBCR data can determine policy makers rules and monitor programs that would affect the GBCI.^12,13^ By analyzing the available data, we will be able to evaluate key strategies to achieve the objectives of GBCI, including health promotion and early detection, timely diagnosis, and comprehensive BC management.^12,13^

 We encountered a number of challenges in our study. Since hospitals and facilities in different provinces have different management systems (Table 1), the primary registry protocol needed to be changed to accommodate the differences. There is missing data in some sections due to these variations across centers. For example, the completeness of staging data was 80.54% for BC in our study, which compares weakly with 94% of the Australian national average or 90% of Canada in 2010 for female BC.^30,31^ In order to overcome challenges, we had to employ a variety of management strategies, including in-person interviews, integrating data from other sources, and conducting telephone follow-ups.

 In conclusion, the information provided by this registry can be used to evaluate and improve the quality of care for BC patients. It will be scaled up to the national level as an important resource for measuring quality of care and conducting clinical cancer research in Iran.
